# "The Hospital" Nursing Mirror

**Published:** 1898-01-08

**Authors:** 


"The Hospital" Nursing Mirror.

Being the Nursing Section of "The Hospital."

[Contributions for this Section of "The Hospital" should be addressed to the Editor, The Hospital, 28 & 29, Southampton Street, Strand, London, W.C., and should have the word "Nursing" plainly written in left-hand top corner of the envelope.]

### News from the Nursing World.

### OUR CHRISTMAS DISTRIBUTION.

Since our last distribution of clothing amongst the hospitals, presented by our readers, another contribution has been received from Madame L. Monchablon and her staff of typewriters. Every year she sends us a delightful box, and that for this year is no exception to the rule. It contains caps, comforters, socks, shirts, and ties for the men; and for women crossovers and stockings. We regret that the box did not reach us in time to send the things out with the rest of the garments, but such suitable and warm articles of clothing are always acceptable.

### THE SCARBOROUGH CHILDREN'S MEMORIAL.

The Scarborough children have placed a beautiful window in the hospital and dispensary in memory of the Queen's long reign. The window, which represents the figures of Faith, Hope, and Charity, has the following inscriptions: "A tear for pity, and a hand as open as the day for melting charity." "This window is the gift of Scarborough children in commemoration of the sixty years' reign of her Most Gracious Majesty Queen Victoria." It was unveiled on the last day of the old year by the mayor (Mr. James Pirie). There were a number of sympathisers present, and the short account given of the Children's League was interesting. Once a month members of the Ladies' Committee go to the hospital to receive the contributions of the children belonging to the Guild, who are then permitted to visit the children's ward. At first the little ones shrank from the sight of the suffering, but now they look upon their visits as a privilege, and the little patients give them a hearty welcome. The children's offerings average £50 a year, and they have this year raised £80 for the window in addition by a sale of needlework.

### ANOTHER NURSES' GUILD.

A new nurses' guild has been formed to promote Christian fellowship amongst nurses who are members of the Church cf England. The lady president is the Marchioness of Northampton, and the medical members of the council are Sir William Broadbent, Dr. C. W. Chapman, and Dr. A. L. Galabin. The secretary is Miss Wethered.

### SISTER MONICA.

Sister Monica, the head nurse of the Warwick Nursing Association, has announced her intention of being received into the Roman Catholic Church, and a considerable agitation for her dismissal has arisen amongst a certain section of the townsfolk of Warwick. The agitation is unreasonable, as the association is nonsectarian, and is supported by subscriptions from people holding every shade of religious opinion. Sister Monica, who is very popular, has given an assurance that she will in no way attempt to propagate her new views. In Warwick the majority of subscribers belong to the Church of England, and in the case of a new appointment the wish of the majority would certainly decide the matter, but the dismissal of a popular and efficient nurse because she has become a Roman Catholic savours too strongly of religious intolerance to meet with much favour with any except those holding extreme views; unless, indeed, there was some agreement on the subject at the time of her engagement. Although the question of creed very rightly is not allowed to enter into nursing questions, yet the diversity of beliefs constitutes a real difficulty. Of course it is pleasant for a patient to have a nurse holding the same religious views as him or herself, but whilst a Protestant may prefer a Protestant, it should not be forgotten that a Catholic equally desires a Catholic, and that neither can always have what he or she wants. The great thing in sickness is to have a good nurse.

### NEWS OF THE NURSES AT THE LONDON HOSPITAL.

The probationers doing staff work at the London Hospital all wear now a silver "S" in order to denote their rank. It appears that their uniforms and that of the ordinary probationers being the same, the difficulty of recognising which was which occasioned a little awkwardness at times, and Mr. Sydney Holland introduced the silver S idea. The London Hospital section at the Victorian Exhibition, Earl's Court, has been the means of bringing the hospital many new friends, and the number of offers from ladies to make garments for the patients must be most acceptable. The multitude of poor patients is appalling, and we admire the courage of those who attempt to grapple with the problems involved in helping them.

### THE NURSES' NEEDLEWORK GUILD.

The institutions which will benefit by the distribution of the first 350 garments contributed by the Nurses' Needlework Guild are the London Hospital, Nazareth House, the Royal Free Hospital, the Clapham Maternity Hospital, the Shadwell Children's Hospital, and the Metropolitan Hospital. The Guild was founded early in the year, and has already achieved considerable success. The first annual meeting was held at 8, New Cavendish Street, and the hon. secretary and originator, Nurse Theobald, announced that she had received the promise of a number of new members for the coming year. The subscription for nurse members is only 6d. (to cover postage) a year and one garment, and for associates 1s. a year and two garments. The object of the Guild is to supply the indigent sick with clothes; and Nurse Theobald, 3, Bolsover Street, W., will welcome all who feel inclined to help in the work, either as members or associates.

### BALLYMENA UNION WORKHOUSE.

Although much credit reflects on the officials of the Ballymena Workhouse from the clean and tidy manner in which both the patients and wards are kept, yet much remains to be done to satisfy modern requirements with regard to nursing and the comforts enjoyed by the patients and attendants. At present there are

90 inmates in the infirmary, of which number 33 are lunatics, and there are only two nurses. The pauper wardsmen and women take their meals and sleep in the wards; whilst the recommendations of Mr. Agnew, the Local Government Board Inspector, respecting this and other matters equally important, have not yet been carried out. The patients have neither knives, forks, nor plates, and additional beds and bedding, lockers, and chairs for convalescents are wanted. Probably much that seems necessity to us is not missed by Irish paupers, but trained nurses must be horrified at having to take charge of 90 patients without the requirements of civilisation, let alone those of nursing. The report shows how very much there is to be done in Irish workhouses before they can be brought up even to the minimum state of efficiency.

### TRAINED NURSES' INSTITUTE, LEICESTER.

The Committee of the Trained Nurses' Institute for the Town and County of Leicester, at their meeting on December 21st, presented a bonus of £2 in addition to the earnings of each nurse in their employ, and added £25 to their sick fund investment account. There are 28 nurses on the private staff and 11 on the district nursing branch. The two branches are kept distinct.

### NEW NURSES HOME AT THE "RETREAT," YORK.

A picturesque building is in process of erection to accommodate one half the nursing staff of the "Retreat"—the well-known Hospital for the Treatment of Mental Diseases, York. Just now very few of the thirty nurses have a room to themselves, they have no dining-room, and have to use the same sitting-room as the patients. The new home will contain twelve bedrooms, a dining-room, and a sitting-room. The removal of these nurses will give more room to the patients and also, it is hoped, secure a continued supply of conscientious women as nurses. The new home is expected to be ready for occupation before the summer.

### THE SOCIAL PRIVILEGES OF THE WORKHOUSE NURSE.

The chaplain of the Kingston-on-Thames Workhouse has lately expressed his disapprobation of male officers calling on and chatting with the nurses in their own sitting-rooms. The Board of Guardians and the master of the workhouse approve, so the chaplain has got nothing for his pains but a snub. Not long ago there was an outcry at one of the workhouse infirmaries because the lady doctor was expected to share the same sitting-room with her male coadjutor. Evidently, therefore, amongst the problems which Guardians have to solve is the extremely difficult task of drawing the line as to the social privileges to be enjoyed by the nurses and officers. We feel inclined to sympathise with the chaplain, for, from an outside point of view, it does not appear desirable for the officers of an institution to be able to drop into the nurses' rooms for a casual chat. It must militate against discipline in some measure, and a little ceremony on the occasion of nurses entertaining visitors is a safeguard against gossip. As to the lady doctor we think she ought to have a private sitting-room, and an equal amount of ceremony is essential to her comfort and reputation.

### SUNDERLAND DISTRICT NURSING ASSOCIATION.

In October last it was resolved to separate the district nursing from that of the Sunderland Institute and affiliate the former with Queen Victoria's Jubilee Institute for Nurses. This has been done, and the rules of the new council and the names of the executive committee were unanimously decided on at the first meeting of the association.

### THE FLANNEL SHIRT CLUB.

The Flannel Shirt Club established in January under the presidency of the Countess of Strafford makes good progress. The praiseworthy aim of this association is to provide the outgoing patients from our hospitals with a new flannel shirt—a very necessary and thoughtful provision, especially in the inclement weather. Subscriptions are gladly received by the hon. treasurer, Mrs. H. H. Chutton, 2, Portland Place, W., and contributions of shirts or materials by the hon. secretary, Miss Lamport, 52, St. John's Wood Road.

### BOSTON CITY HOSPITAL.

A timely gift of about £20,000 has been received by the Boston City Hospital from the Ann White Vose estate for the purpose of constructing an additional nurses' home. The training school attached to this hospital has a growing reputation, and a larger number of more eligible candidates apply yearly. The course of lectures, cookery demonstrations, clubs, and libraries, are numerous and evidently popular. The total number employed last year was 129.

### BELFAST NURSES' HOME.

The annual report shows that there are ninety nurses on the staff of the Belfast Nurses' Home and Training School. The past year has been a busy one, and there is a substantial balance to the credit of the home. There have been heavy expenses in repairs and refurnishing, but the building is now in order. In spite of the large staff, 145 cases have had to be refused.

### BURTON-ON-TRENT INFIRMARY COLLECTION.

A larger sum than ever has been collected—viz., £1,179—on Infirmary Saturday by the townsfolk of Burton-on-Trent. Of this, £100 has been given to the Nursing Institution. We are glad to see that the Jubilee contributions have not affected the permanent charity of the district. This is as it should be. If the celebrations of Her Majesty's glorious reign meant simply transferring the sums usually sent to established charities to new ones, they would have harmed rather than helped the cause of the poor and suffering, a circumstance that could afford little satisfaction to anyone, least of all to the monarch in whose honour they were held.

### SHORT ITEMS.

A district nurse has been appointed to the parish of Kilbrandon and Kilchattan (Oban) through the kindness of the county secretary of the Queen's Jubilee Institute for Nurses, the Duchess of Argyll.—Penryn and Gluvias Nursing Association became affiliated to the Queen Victoria's Nursing Association in March last, and have now the benefit of a trained nurse who gives great satisfaction. The receipts amounted to £51 and the expenditure to £39. Efforts are to be made to increase the subscriptions as the present nurse has a higher salary than the last. We hope the committee will remember that no district nurse ought to be asked to live on less than £60 a year with lodgings found.—Dr. Wade has had a cheque of £60 forwarded to him for the Torquay Nurse Institute by "one who knows the value of nurses for the sick poor."

### Practical Aspects of a Nurse's Life.

By Sister Grace.

### XIII.—SISTERS.

### Qualifications Needed.

In speaking to nurses I wish it to be distinctly understood that I in no way seek to instruct those who are probably quite as experienced as myself, but to address a few friendly words to beginners in the life; it is not an easy one, and when first made a sister myself I should have been most thankful to anyone who would have given me a few hints. It is difficult to find suitable women for ward sisters, because they need to combine in themselves so many different qualifications. Failure is not uncommon, because a staff or night nurse, promoted on account of her success in her own department, has not always the power of organisation. She could faithfully obey, and see that others obeyed orders, but she cannot organise. Then, again, tact is indispensable—the amount of this valuable gift needed to make things go smoothly in a ward is stupendous when you think of the different tempers and dispositions of some thirty patients, of the numbers of physicians and surgeons to please, in spite of the many petty jealousies and heart burnings that, alas, are not absent from hospital life; of the different house surgeons, who must never be allowed to feel that one is more considered than another; when you remember the well-meaning, but often intrusive, visits of wealthy subscribers, and the still more intrusive questions and catechism of district visitors, who seem to imagine they have a right to visit the patients at all hours and under all circumstances; when again you recall the ministers of religion of all denominations, who also feel aggrieved if they cannot visit in season and out of season, and thousands of other difficulties, out of which (as subordinates) we slipped with a comfortable sense of no responsibility, I am sure you will see that unless a woman has immense tact, unfailing patience, and self-control, she had better not aspire to the honourable, but very onerous, position of a sister.

### Attitude to Doctors.

You will agree with me as to the necessity of giving the most accurate and careful attention to everything that the doctors say; but I want to speak specially of your behaviour with the house physicians and surgeons. They are generally young, and, like the rest of us at that stage, sometimes a little inclined to carry things with a high hand, and to assume a tone with the sister as of one speaking from the heights of Socratic wisdom to a beginner treading the thorny path of the alphabet. I do not think this is often the case. I can recall many kind and valued friends among house surgeons, and the useful help and information that they were invariably ready to give me; but I know such cases exist sometimes, and you will, I think, entirely disarm them by observing towards them an attitude of entire respect and attention.

Like sisters their life is a difficult one, and their tempers are often sorely tried; see that, at any rate, they are not further exasperated by anything that you can avoid. The comfort and well-being of a ward largely depends upon whether the house surgeon and sister work well together or whether they swim in different currents. This will rest chiefly with the sister. Never assert your own opinions and wishes, but defer to his, and you will find that in the end you generally have your own way. It is always easier to lead than to drive. This is a truly feminine piece of counsel, and I beg you to lay it to heart. In medical wards there is much note taking, which sometimes is wearisome, especially in the women's wards, where there must generally be a nurse in attendance; but if it is so to you it is far more so to the house physician. Do not be irritable if he comes unavoidably at an awkward time. Rules must be observed, but a pleasant word and smile robs a refusal of its sting.

### Interviews With Patients' Friends is a duty that presses heavily on the sister, especially in operation and accident wards. It is difficult to speak the strict truth without, perhaps, alarming them unduly, and they generally repeat the conversation to the sufferer, enriched by their own dismal prognostications. They are usually intent upon getting your own opinion as to the advisability or the reverse of impending operations. I need hardly warn you not to fall into the snare thus spread for you, but to steadfastly refuse all expression of opinion and refer them to the doctor. But you can often afford them some degree of comfort by explaining the state of the patient to them in very simple language; poor things, they understand so little of the technical terms used by the doctors. And in cases where you must see the friends of a patient so seriously injured as to preclude all hopes of recovery, or when death has already taken place, I need hardly say that here is an occasion to call to your help all the womanly tenderness and sympathy you possess.

Let me here remind you of the sad duty of giving the last attentions to those who have passed away. When I began my nursing life I was so distressed by the callous manner in which this was sometimes undertaken, that I determined, if ever I became a sister, I would always assist in those duties myself, and impress by all means in my power on my nurses the duty of displaying to the dead all the reverential tenderness and delicacy possible. Remind your nurses what they would feel if one dear to them died in hospital, and was treated otherwise than with refinement and respect. It is always a painful duty, and it recurs so frequently that it is necessary, especially with young nurses, to invest it with as much solemnity as possible, to guard against the danger of familiarity breeding contempt; and it is a good plan, if possible, to be yourself present when the porters come to fetch away the dead. Without intention, simply from want of thought, their manner is often very careless, and it has a good moral effect on them to find the Sister there, paying what respect is possible to what has been the earthly habitation of an immortal soul.

### An Accidental Death.

Miss Rose Murden, a trained nurse, met with a sudden death whilst attending a case in the family of a solicitor in Leicester. It appears she had gone to the larder and struck a match, which immediately caused a loud explosion. The drawing-room floor was torn up, the furniture wrecked, and the unfortunate nurse had her skull fractured. The occurrence is attributed to a gas leakage, but it seems strange, if this were the case, that the victim should not have been warned by the smell. Can it be possible, after so many terrible accidents from this cause, that it is still necessary to tell people that they must not look for a gas escape with an open light? Nurse Murden has paid very dearly for her carelessness, poor girl.

### Where to Go.

North-Eastern Hospital for Children.—Christmas Tree on Wednesday, January 12th, from half-past three to six p.m.

Metropolitan Hospital, Kingsland Road.—Entertainment for the patients on Friday, January 7th, from four to half-past seven p.m.

### Post-Graduate Clinics for Nurses.

By a Trained Nurse.

XXXIX.—THE NURSING OF TYPHOID (*continued*).

It would not be right to conclude the dieting of typhoid fever patients without saying a few words as to the drinks—apart from nourishment—which they will need.

I confess to being a little tired of the toast-water and Potus Imperialis, which figures so largely in invalid cookery books. Many other drinks are much more pleasant. Where high fever, parched mouth and throat exist, mucilaginous drinks quench thirst effectually, for these form a mucilaginous coat on the mucus membrane which prevents evaporation. Calves' foot jelly, thin enough to remain fluid when put on ice, is a refreshing drink—isinglass being preferable to gelatine, unless the fresh calves' feet be used. Hot barley-water poured on to fresh lemons or fresh oranges, and slightly flavoured with some good sherry, is very pleasant, as are also lemonade and soda water mixed in equal quantities. Champagne and soda water mixed are admirable. Teaspoons of cold tea or coffee quench thirst well without bulk. And the juice and rind of half a lemon, with a small piece of bruised ginger and three-quarters of a pint of boiling water or barley water poured over, sweetened with grape sugar, strained, iced, and flavoured with good sherry, is a delicious fever drink. Some doctors allow typhoid patients to take the expressed juice of fresh grapes, care being taken that no seed or skin finds its way into this wholesome and refreshing beverage, to which a little soda water may be added. I shall have something to say as to convalescent diet when our patient reaches that desirable stage.

Meanwhile, something may with advantage be said of the duties whereby a nurse may guard against the outbreak of "secondary cases," which if she be not watchful may arise either in the household of the patient or in the community of which he is a member. I spoke in a previous paper of the necessity of allowing the excreta to soak for some ten minutes in a disinfectant—such as sulphate of iron, strong carbolic solution, or perchloride of mercury. Many nurses who carefully carry out this precaution forget that the typhoid germs appear in considerable numbers in the urine, and that this, too, needs treatment with disinfectants. The typhoid germ is very powerful if allowed to grow outside the body, as on linen, &c. And it should be remembered that sprinkling with a disinfectant has very little value. Totally submerging the linen is the only plan to safely pursue. Nurses should always impress upon private patients the desirability of using their second best body and bed linen in typhoid and other infectious cases. For though the nurse exercise a superhuman cautiousness, disinfection necessarily means deterioration. No flannels and blankets can preserve their excellence when put into boiling water, yet this is essential to complete disinfection. If the water be really boiling, and be kept boiling, disinfectants need not be used. And it must always be borne in mind that it is essentially the duty of the nurse, and not of the parlourmaid, to rinse through disinfecting fluid all the linen of her patient, and thus see that this is rendered perfectly safe for sending to a laundry. If the house contain the old-fashioned comfort of a copper, disinfecting by boiling will be an easy matter. Otherwise science tells us that linen needs soaking for two days in a 1 in 1,000 solution of bichloride of mercury. This is described, from the medical point of view, as death to the microbe. Housewifely nurses know that bichloride of mercury means death to the linen, by discolouring and roughening it. If boiling the linen can be arranged for, the milder disinfectants may safely be employed.

It is part of a nurse's duty to influence the household in which she is nursing a typhoid case, to see to the flushing and disinfecting of w.c.'s, drains, and remind them of the value of boiling all the milk used by the family. She cannot, of course, force theories on people who do not care for health, but with a little tact and judgment may point the moral of "secondary" danger. And the fact that she allows none but sterilised or boiled milk for her patient will carry a salutary lesson. In caring for a case of typhoid, I invariably undertake the boiling of the milk for the patient—and apparently from good nature, but really with a view to public safety, frequently offer to include the milk used throughout the household in my prolonged cooking. For the "boiling" of the average cook is a merely perfunctory simmering. To avoid burning and waste, the best method is to place the milk to be boiled in a clean tin vessel. Fix this in a cauldron of water and keep it at boiling point for half an hour.

The nurse must protect herself from being the first "secondary" case—if one may so express it—by carefully avoiding exhalations from the patient's excreta and urine, and particularly by the occasional use of a Condy mouth-wash. Her nails should be kept very short, a soft nail-brush used frequently, and hands be dipped often in a basin of disinfectant. Nurses may here be reminded to choose for their personal use one of the several disinfectants which do not coarsen and roughen the skin of the hands. For it is not only important from the æsthetic point of view that a nurse should carefully keep her hands soft and smooth. It is so much more pleasant to the patient to have the contact of nice hands. And as every nurse soon realises, unless she take some little trouble in this direction, rough ward work and constant washing and dipping in strong antiseptic solutions, both at operations and clinically, will soon work havoc in the most dainty hands.

### Appointments.

### MATRONS.

The Hospital Samaritans, San Paulo, Brazil.—On December 18th, 1897, Miss Lillian Lees was appointed Matron of the above hospital. She was trained at the Manchester Children's Hospital and King's College Hospital, London. She has held appointments as ward sister at the Lewisham Infirmary, and was one of the nursing sisters at the late war in Greece.

### Minor Appointments.

The Northern Convalescent Hospital, Winchmore Hill, N.—Nurse E. M. E. Heys has been appointed Night Superintendent of nurses at this hospital. She was trained at the Mill Road Infirmary, Liverpool, and for the past two years has been charge nurse at the hospital ships, and also at Gore Farm Hospital.

Joint Isolation Hospital, Cuddington, Surrey.—Miss J. Roose was appointed Sister of the above hospital on January 3rd, 1898. She has been previously sister at the Children's Hospital, Birmingham, and staff nurse at the Children's Hospital, Great Ormond Street, London. She was trained at University College Hospital, London.

Luton Union Workhouse Infirmary.—On the 3rd inst. Miss Elizabeth S. Owen was appointed Superintendent Nurse of this infirmary. She has previously held appointments at Toxteth Park Hospital and Sheffield Union Infirmary, and was trained at Liverpool Royal Infirmary Training School.

### Christmas Entertainments.

BROMPTON CONSUMPTION HOSPITAL.—On Thursday an enjoyable entertainment was given in the concert room at the Brompton Hospital for Consumption to the patients, at which many friends and well-wishers of the institution were present, amongst whom were Mr. T. C. Beckwith (chairman of the Committee of Management), Colonel the Hon. H. Eaton, General Trotter, Mr. H. R. and Mrs. Chapman, Mr. George Rose, Mr. Gerard Young, Mrs. Barnard Smith, Miss Davidson (the matron), Mr. Theobald (secretary), the Rev. H. N. G. Hall (chaplain), Dr. Falkin (resident medical officer), and Dr. Brownrigg, who took the part of Father Christmas. The first part of the evening was devoted to stripping the tree of its numerous gifts. The nurses stood round with outspread aprons and then distributed their spoils to their own special patients. The body of the room was filled with inmates, and most of them held on their laps substantial brown paper packages containing warm garments chosen to suit each recipient's particular need. After the tree came the entertainment of conjuring and ventriloquism. Light refreshments were served in the hall and were much appreciated.

MOORFIELDS EYE HOSPITAL.—On December 31st an excellent musical programme was gone through for the amusement of the patients of this hospital. The Rev. Prebendary Whittington, chaplain, took the chair. Mr. Hogg, the chairman of the House Committee, and many members of the surgical staff were present. The out-patient department was tastefully decorated with foliage, fairy lamps, and Chinese lanterns, whilst screens and curtains were placed to produce a good effect. This was the work of the matron (Miss Robinson), assisted by her nurses and the resident medical officers. The Boracic Brothers and the Variety Trio, from the London Hospital, kindly gave their amusing entertainment, which was heartily applauded, and some of the audience added to the "go" of their songs by joining in the refrains. Miss Fisher's violin and Mr. Carr's banjo was clapped vigorously and the two lady vocalists, Miss Giblett and Miss Buckland, both received encores, to which they kindly responded. At the close the Chairman proposed a vote of thanks to the performers, which was seconded by Mr. Dunn. This gentleman paid a high tribute to Miss Robinson, the matron, and referred in warm terms to the excellent work she had done since her appointment as matron. His words met with hearty applause, which must have been most gratifying to the lady. Refreshments were served later, of which most of the guests partook. The Christmas tree had been given the evening before, and held gifts for all. It was greatly enjoyed by old and young alike. One old man from the country declared that he had never expected to see such a beautiful sight in his life.

ST. MARY'S DAY NURSERY AND CHILDREN'S HOSPITAL, PLAISTOW, E.—The inmates of the St. Mary's Day Nursery and Children's Hospital at Plaistow, E., had their Christmas entertainment on Thursday, December 30th, and many friends took this opportunity of inspecting the institution. The building was prettily decorated with ivy, flowers, lanterns, and fairy lamps, the two hospital wards especially being much admired. The little patients had taken a lively interest in the progress of the decorations for days before, and quite looked upon it as their own treat. After tea, all the patients who could not walk were carried down to the play-room of the nursery, where, with the regular attendants of the day nursery, they witnessed a Punch and Judy show. "Father Christmas" next appeared, and distributed the gifts from a giant Christmas Tree, which, covered with toys and glistening with lights, had been a source of great attraction to the children. The tree itself and all the toys were given by friends of the institution, and included some sent by "Truth." Among the visitors were the Archdeacon of Essex (the chairman of the committee), the Rev. T. Given Wilson (vicar of the parish, and founder of the institution), nearly the whole of the medical staff, and many subscribers to the special named cots. The day nursery has accommodation for between 30 and 40 children, and 4,534 have attended during the year. The hospital now contains 30 beds, of which 6 are in the isolation cottage, and the average number occupied daily during the past year has been 26. The in-patients have numbered 479, of whom 410 were admitted into the hospital and 69 into the isolation cottage. The out-patients have numbered 2,461, and the casualty cases 3,623.

### Queen Victoria's Jubilee Institute for Nurses.

HER MAJESTY has been graciously pleased to approve the appointment of the following as "Queen's" nurses, to date January 1st, 1896 :—

ENGLAND.

SUPERINTENDENTS.—Ann I. E. Phelps, Hampshire County Nursing Association; Mabel Rogers, Sunderland.

NURSES.—Edith Hughes, Stamford; Agnes H. Hanning, Hammersmith; Margaret E. Trinder, Chelsea; Alice E. Bullen and Agnes Driver, East London; Hester F. Levis, Bradford, Manchester; Clara Sharpley, Ardwick Green, Manchester; Helen Robinson, Alice Trotter, Charlotte E. Browne, Mabel Clarke, and Olive S. Walters, Salford, Manchester; Jane Allen, Grimsby; Ada K. Treasure, Bolton; Marian Porter, Hannah Harris, Emma Young, and Margaret Fisher, Birmingham; Jennie Jones, Coventry; Ada M. Holmes, Bedworth; Eliza J. Campton, Mary Taylor, and Ada Price, Northampton; Fanny Sheldon and Florence N. Shore, Reading; Isabella Stephenson, Penzance; Charlotte L. Hedderly, Leeds; Esther C. Bell, Woolwich; Annie Cooke, Hanley; Gertrude Fisher, Bishop Auckland; Annie Taylor, Leamington; Mary Gaskell, St. Austell; Jessie Jackson, Torpoint; Ada L. Wood, Grantham; Sarah Repton, Louth; Mary E. Elwell, Southwell; Mary A. C. Littlewood, Thrapston; Amy Chadwick, Newmarket; Annie Baynes, Loughton; Elizabeth Wilson, Castor and Sutton; Eliza Lawton, Lytham; Elizabeth I. Barr, Hebden Bridge; Rachel Parsons, Liversedge; Sarah L. Vergette, Warrington; Janet Anderson, Buxton; Sarah H. Broadbent, Chorlton-cum-Hardy; Helen Ferrer, Bath; Edith Butler, Weston-super-Mare; Mariabella Fry, Long Ashton; Edith M. Allen, Highcliffe; Edith A. V. Stephens, Love; Alice Adkins, Aldershot.

WALES.

NURSES.—Mary C. Jones, Carnarvon; Mary White, Llanrwst; Alice Edwards and Elizabeth Edwards, Festiniog; Florence Kewley, Llandaff; Mary Scott-Wilkinson, Oystermouth; Edith E. Please, Neath; Bessie Barter, Neyland.

SCOTLAND.

NURSES.—Grace Strange, Edinburgh; Anne J. Little, Blairgowrie; Grace F. Campbell, Aberdeen; Kate Porter, Stewarton; Margaret Sinclair, Port Glasgow; Bathia Rennie, Uddingston; Elizabeth Fisher, Stornoway; Priscilla Paterson and Annie G. Buchanan, Wick; Elizabeth Mackenzie, Glenurquhart; Janet Carstairs, Sara M'Callum, and Elizabeth S. D. Tralan, Tobermorey; Janet Turner, Glasgow; Marian Macquarie, Paisley; Emily Holden, Dundee.

IRELAND.

NURSES.—Mary Montgomery, Edith Peile and Catherine Young, Dublin; Mary J. Lee, Achil Island; Rebecca S. Pinnions, Coleraine; Frances J. Walpole, Blackrock, Stillorgan, and Booterstown; Margaret Wallace, Carrickfergus; Kathleen F. R. Norcott, Portadown; Kathleen M'Auliffe, Ballymena; Annie M'Keown, Armagh.

### Nursing in Italy.

By a Correspondent.

In view of the recent discussions as to the advisability of nursing by nuns, it will be interesting to glance at the results of the system in Italy, where the nursing arrangements are almost entirely under the charge of religious sisterhoods. The first thing that strikes the eye of an English nurse is the apparent indifference to personal cleanliness that prevails. Having been accustomed in an English hospital to think that a clean skin for the patient is one of the first conditions of good nursing, she finds it hard to conceal her disgust that sick people seem oblivious of the necessity for even the most moderate daily wash.

A woman who had passed several months in a large hospital told me that it was only by dint of the most urgent entreaties that she could procure a basin of water and a towel every morning, and then she was usually indebted to the kindness of a convalescent patient. She had been a servant for some years in an English family, which accounted to the other inmates for her extraordinary desire to wash every day.

It is the same in private nursing. I went to see a lady who had been ill with influenza for nearly three weeks. During this time she had neither washed her face nor done her hair; and, unfortunately, on the day after she began the use of soap and water once more she had a relapse, which was at once put down to her foolhardiness in not waiting till she had fully recovered.

In most of the hospitals the sisters do not undertake the details of nursing themselves. There are a number of women called *infirmiere*, who receive a sort of haphazard training, and who attend to the sick in a general way and keep the wards clean. These women sometimes have a uniform and wear caps and aprons, but often they wear the dress of the peasant classes, including wooden-soled shoes, in which they go clapping about the stone floors. They are very kind to the patients, and obtain a good deal of experience in attending to the sick, but in our sense of the word they are certainly not nurses.

The sisters look after the ordering of the hospital and attend to the dispensing of medicines and at operations. They belong, as a rule, to the various Orders of St. Francis, and wear dresses of either white or brown cloth. They seem to belong to all classes, but many of them are ladies by birth, and are educated and intelligent women. They have a passive sweetness about them which is very soothing to weak and irritable nerves. On the other hand, to those patients who want a moral stimulant, their calm acquiescence in the "Will of God" theory, as expounded by Tennyson's "Farmer's Wife," is extremely depressing.

Though some of the newer hospitals are beginning to adopt the modern theories of sanitation, yet many of them are very behindhand in this respect. Bath-rooms, water closets, drainage systems, will not compare with those in use in England and Germany. In all matters of hygiene, besides personal cleanliness, things are as bad as they can be. Disinfectants are used, but their real worth is imperfectly realised by the *infirmiere*; she values them as saving the labour of thorough cleansing. For this reason the utensils in use by the sick are usually smelly and unpleasant. The bed pans and urinals are used by the patient and then put under the bed until the attendant comes round. In the night, when the patients are sometimes left for several hours at a stretch, the atmosphere of the wards is decidedly unwholesome.

As a rule the Italian hospitals appear exceedingly well ventilated. This is not due to any system, but rather to the fact that space is of little value, and that therefore the wards, corridors, and offices are lofty and spacious. The immense whitewashed rooms have very little furniture beyond the rows of narrow beds, which are hard but not otherwise uncomfortable. Floors, tables, and bedclothes appear very clean and fresh, this part of nursing being better understood than the personal. Everything looks bare and unhomely when compared with our own hospital wards. The eye longs for a vase of flowers or a pot of evergreens to break the monotony of the long rows of beds and big white walls; but that the ward or sickroom should be made pleasant to the eye has apparently never entered anybody's mind.

Night nursing generally consists in making the patient comfortable at an early hour of the evening, and telling him to shout if he wants anything. In the paying wards which are attached to some of the hospitals the sick are provided with hand-bells. In most cases the sisters retire at night to their own quarters, but one or more of the *infirmiere* either sit up or sleep in rooms near the wards.

Visitors are admitted every day, usually for several hours. There appears no restriction as to number, and whole families of excitable people come and have a jubilant time round the beds of their sick relatives. I used to think that the chattering, gesticulating, and even quarrelling that went on must be very bad for the patients, but two years' experience of the Italians has convinced me that their excitement is only skin deep, and they quickly subside into calm when the visitors go.

In the common wards there is the usual rule that no food shall be brought to the patients by their friends, but it is not very strictly enforced. I was one day in a large hospital when a woman brought her sick husband some most unwholesome-looking cakes. The *infirmiere* remonstrated. "Do you want kill to him?" she asked the wife. "The Madonna will take care of him," the peasant woman said, piously. That seemed to be answer enough; the patient was left in possession of his cakes, though he looked more fit to be milk diet. In the paying wards the patients can have food sent in by their friends.

The science of feeding the sick is very little understood Low diet consists of milk, raw eggs, and weak broth sometimes thickened by maize-flour. The convalescent has various dishes of macaroni and rice flavoured with grated cheese; meat is allowed, but it is hard and unpalatable. In private nursing a standard invalid dish is a custard made of yolk of egg and Marsala wine. It can be served as a pudding course or eaten as an extra between meals. The taste is pleasant, and it is a stimulating and nutritious food for a weakly convalescent.

The impression left on the mind is that the religious training of the nuns prevents them from making good nurses for severe cases. In chronic cases and in the diseases of old age their patience and kindness make them delightful sick attendants. Their lessons of submission hinder them from bringing determination to bear in the fierce struggle with death that occurs in more severe diseases. The saying that it is good to live but better to die is a bad one for a nurse.

### CHRISTMAS DAY AT CHELSEA HOSPITAL FOR WOMEN.

Christmas Day at the Chelsea Hospital for Women was generally pronounced by the patients as the happiest they had ever spent. They were allowed to entertain their friends at tea, and the nurses gave them great pleasure by their charming decoration of the wards, and by their carol singing in the evening.

### "She Hath Done What She Could."

By SISTER OLIVER.

THERE are a number of books at the present day pointing out various and essential qualities a woman ought to possess to enable her to become a good nurse. They are bought and read, and many doubtless try to follow the rules and advice given. If a nurse loves her work and is anxious it shall become a success she will read all the helpful literature and attend as many lectures as she can. Such advantages cannot be too highly valued. After several years of hard work and close study some may be able to step to the front and become "matrons" or "sisters," whilst others have to content themselves with working in the shade; but let not such become discouraged at not holding the same position as their more fortunate sisters, and think all the time devoted to our work wasted. No time is wasted that is spent for God.

Success is sweet, but to many dispositions it is most injurious. One is apt to become self-satisfied, and to think that, having reached the height of one's expectations, there is nothing more to be done but to fulfil one's duties and enjoy life as much as one can. That is all very well from the world's point of view, but not from God's. He wants those talents devoted to His service. The influence of a fully consecrated life (though not always seen) is immense and incalculable. If every nurse would only consecrate her life to God, and do all her work as His minister, how much easier that work would become, and what a different spirit would pervade, not only the hospital but every house she entered.

The words, "She hath done what she could," do not mean the number of certificates she may hold or the number of big operations she may have witnessed; neither do they mean getting up early in the morning to attend celebration, and, perhaps, in consequence of being very tired, speaking sharply to some patient who may ask her to perform a little service on her return. It means every little service rendered cheerfully—"the cup of water given in My name," the little words of comfort, and the prayers she can offer up for the poor sufferers as she goes about her work.

Someone may say, "Oh! we've the chaplain," or "the clergyman visits the house; that, surely, is his work!" True; but if all are working for the "Great Physician," ought not the same spirit of interest to be at work with one and all alike?

I do not mean that religion is to be thrust upon the patients; that would be a very grave mistake, and one which many would deeply resent; but there are times when a few words spoken in "season" are priceless. The point to be brought forward is this—that although it is impossible for all to become clever, yet all can be good. And if only a little more genuine sympathy were thrown into the everyday work, and if each nurse would only show the same care and thought for the comforts of her patients as she often exhibits in leaving the wards at an appointed time, what a haven of rest the hospital and sick-room would become.

A clergyman once remarked in the course of conversation that it was very sad to think how few of the vast number of nurses professing Christianity are so in truth, and are, therefore, conscious of the great responsibility resting upon them or of the many opportunities they have of being able to give spiritual comfort. He went on to relate how for several weeks he had tried to gain admittance into a certain house where a woman lay dying, but without effect; so all he could do was to pray to God that her nurse might be one who knew the power of Divine love. But, he added, she may be one of the many who think their duty fully accomplished by giving the medicine correctly and taking the pulse.

Surely there is a great fault somewhere; is it because one gets so wrapped up in a little world of one's own, that it becomes impossible to have a thought or care for anyone or anything outside one's self? Or is it through neglect of prayer? I do not mean using a set form of words every day, but to hold sincere intercourse with God. Have we ever tried to find out the duties our heavenly Master expects from us? Our Lord not only ministered to the bodies of the sick, but to the whole man, body, mind, and soul; and we ought to endeavour, as far as possible, to do the same.

The mistake that so many make is this: They choose someone—say a Florence Nightingale or a Sister Dora (would that there were many such practical, energetic women in the world)—who realises their ideal of what a woman ought to be, set her on a pedestal, and in trying to shape their own character after such an one, they quite lose sight of that Divine life which alone can teach them the patient endurance, the complete surrender of self, and the Christ-like ministry that must make their lives a comfort and blessing wherever they go. A nurse must above all things be in earnest, must put her whole heart and soul in her work, must go about it as if she meant it; she must never give people the chance to say such things as "My nurse's thoughts were anywhere but on her work," or "All my nurse seemed to think about was flirting or empty novel-reading."

These things are not nice to hear, but the truth must be faced, however unpleasant it may be. Let nurses, therefore, one and all search their hearts, and part with anything that hinders a complete consecration; let them give all time, energy, and talents to God, and then they will not have any moments to fritter away in frivolous pursuits. And when the time comes for them to lay it down, may that work be perfect, yet simple, so that another sister may be able to pick up the threads and continue it. They will then have the comforting assurance that they have not only fulfilled their duties to their fellow creatures, but also to their heavenly Father, and will be rewarded by hearing those blessed words, "She hath done what she could."

### Presentations.

EDINBURGH TRAINED NURSES' ASSOCIATION.—On Christmas Day the nurses of this association presented Miss Thistle, their lady superintendent, with a handsome silver kettle and spirit lamp. Since the opening, a year ago, of this association, which is worked on co-operative lines, there has been no doubt of its success. Miss Thistle, who has worked unremittedly on her nurses' behalf, is to be congratulated on the result of her labours.

As Father Christmas passed through the gaily-decorated wards of the Birmingham Children's Hospital, he stopped by each cot and left a charming gift. Friends and the *Daily Mail* Christmas Tree Fund had provided trees in every ward, and before the venerable Father took his leave he presented the matron, Miss A. J. Lloyd, with an épergne on behalf of her nursing staff.

ON New Year's Day the nurses at the City Hospital, Walker Gate, Newcastle-on-Tyne, presented Miss Ogston, the matron, with a handsome biscuit box as a token of their esteem.

### Wants and Workers.

MISS BROWN, 7, St. George's Place, Canterbury, would be glad to know if there is any home where a young man of 21 could be received. He is of weakly intellect, but willing and able to work under supervision. He has no means.

CAN anyone recommend me an institution where the following case would be eligible for admission? An orphan boy, age 17, without any means, one side paralyzed since birth, but able to go about. He is not an imbecile, but simple-minded, consequently he has been unable to learn.—Nurse Margaret, 4, Hospital Gardens, Oxford Street, Manchester.

WOULD any reader of the "Mirror" care to change a medical book on "Drugs and their Uses" for "Nursing," by J. A. Hampton. The book was bought last October, and is in good condition, price 10s. 6d.?—Nurse P., Dderio, Rhayader, Radnorshire.

### A Book and its Story.

"THE WAYS OF LIFE."

THE death of Mrs. Oliphant has left a gap which will not be easily filled. The time has not yet come for attempting to form an estimate of the place which this amiable and accomplished woman will finally hold in the history of British literature, but there can be no doubt that from the first her success as a writer was assured, for she combined, in a remarkable degree, a facility of expression with an indomitable industry. It is as a novelist that she is best known, though as a writer of history, biography, articles in *Blackwood's Magazine* and other periodicals, and as the editor of *Foreign Classics for English Readers* she produced enough good work to have made a lasting literary reputation. And now her last work lies before us. This volume contains two short stories, tinged deeply with the sober colouring of "the clouds which gather round the setting sun." Of the sorrows of life Mrs. Oliphant had her full share. All her friends knew that the death of her husband within a few years of her marriage, followed by the loss of her sons, had driven the iron into her soul. The aching heart was there; and, as if the sombre thoughts could no longer be restrained but must find utterance, nothing can exceed the tragic pathos of the book which she has named "The Ways of Life."* Sadder, perhaps, and certainly more suggestive than the stories themselves, is the preface, in which she speaks of that phase of human experience which forms the subject of her work—the ebb-tide in the affairs of men. "The moment," she writes, "when we first perceive that our individual tide has turned is one which few persons will find possible to forget. We look on with a piteous surprise to see our little triumphs, our not little hopes, the future we had still believed in, the past in which we thought our name and fame would still be to the good, whatever happened, all floating out to sea to be lost there, out of sight of men. In the morning all might seem as sure to go on for ever—that is, for our time, which means the same thing—as the sky over us, or the earth beneath our feet; but before evening there was a different story, and the tide was in full retreat, carrying with it both convictions of the past and hope in the future, not only our little laurels, all tossed and withered, and our little projects, but also the very heart of exertion, our confidence in ourselves and providence. No rebellion against fate," she points out, "is of the slightest use to the man from whom the joy and pride of life has departed. He must go on, as if nothing had happened, 'in a cheerful despair,' and receive, without revealing the dreadful secret, the compliments of friends and the commonplace amenities of social life. What in the jargon of the age is called the psychological moment is that in which the first discovery is made, and the startled victim suddenly perceives what has happened to him, and feels in every plank of his boat the downward drag of the ebb tide, and looks about him wildly to see if there is anything he can lay hold of to arrest it, any deliverance, or any escape." Mr. Sandford, the subject of the first story, is an artist, a man approaching sixty, who had, for a number of years, made a considerable income by the sale of his pictures. He has a wife, who is devoted to him, and a family of grown-up sons and daughters. "They were all full of faculty, both boys and girls, but all took a good deal out of the family stores without bringing anything in." There was never any apparent want of money. Their father's income supported them in comfort. Everything went on happily. But suddenly Mr. Sandford found that his popularity as a painter was waning. He failed to obtain commissions. His pictures would not sell. He had ceased to be the fashion, and, as the precarious earnings of his profession formed his only source of income, it was evident that poverty stared him in the face. Of all this his wife and children knew nothing; and then a dreadful temptation, born of the love he bore his family, assailed him. His life was insured so that, if he died, each of his children would receive a portion of £1,000; and, in the solitude of his studio—for the rest of the household were enjoying themselves at the seaside—he began to reflect that his death would solve the problem. Still, he could not do it, and "Unless, by the grace of God, something should happen—that was what he kept saying to himself when he reflected on the disclosure which must be made when the seaside season was over." And something did happen. He went to pay a visit in the North. There was a carriage accident, in which he received fatal injuries. "After doing his best for his own, and for all who depended upon him in his life, he did better still, as he had foreseen, by dying." After his death his pictures sold, his daughters married, his sons prospered, and his wife was consoled.

The second story, "The Wonderful History of Mr. Robert Dalyell," is that of a man on whom misfortune comes suddenly, and who deliberately plans to save his family from ruin by disappearing suddenly, in such a way as to make the world believe that he has been drowned. "The condition of the man," says Mrs. Oliphant, "who blots himself out of life without dying, and accepts a kind of moral annihilation while yet all the sources of life are warm within him, might well afford us one of the most tragical chapters of human history." We must refer our readers to the volume itself for the plot of this second story, which is so highly sensational as to be almost impossible. The characters, however, especially that of Patrick Wedderburn, the old Edinburgh lawyer, are drawn with all Mrs. Oliphant's wonted skill; and it is with a feeling of unfeigned regret that we lay aside the last production of the venerable authoress. One thing is certain—that, while the world of letters has lost an ornament, those who knew her have lost a much-loved friend, and none of them will soon forget how much of what was generous and good and kind lies buried in the grave of Margaret Oliphant.

* "The Ways of Life." Two Stories. By Mrs. Oliphant. (Smith, Elder, and Co., London. 1897.)

### Everybody's Opinion.

**[Correspondence on all subjects is invited, but we cannot in any way be responsible for the opinions expressed by our correspondents. No communication can be entertained if the name and address of the correspondent is not given, as a guarantee of good faith but not necessarily for publication, or unless one side of the paper only is written on.]**

### OUR CHRISTMAS DISTRIBUTION.

MISS MARY HULL, the matron of the Great Northern Central Hospital, Holloway Road, N., writes: Please accept our best thanks for the very acceptable garments you so kindly sent us. We had a most enjoyable Christmas, and are very grateful to all the friends who helped to make it so by their kind contributions.

MISS M. R. EASTON, matron of the Royal Hospital for Children and Women, Waterloo Bridge Road, S.E., writes: I beg to tender you my sincere thanks for the most acceptable parcel of clothing which you have kindly sent to this hospital from the readers of THE HOSPITAL journal. I can assure you we shall find the vests and petticoats most useful, and we are very grateful to those who have so kindly remembered us.

MISS BLOMFIELD, resident lady superintendent, East End Mothers' Home, 396, Commercial Road, London, E., writes: Please accept my very sincere thanks for your kindness in sending such a useful parcel of clothing for my patients. I do think it is so very kind of you to remember this little home in your charity.

### EGYPT'S NEED.

"An Old Egyptian Resident" thus writes: There is plenty of opening in Egypt for good works if they were taken in hand in a really practical way. The richest of the English-speaking population come here every winter, but they have money to spend only on their own amusements. While there are French, Italian, German, Austrian, and Russian charities in Cairo, there are no English ones, and this, too, after sixteen years of occupation. Even sisters of mercy have not yet found their way here. We have a few hospital nurses, and that is all.

### TENDER FEET.

"A Nurse" writes: May I be permitted to give your correspondent, Agnes Lakeman, the benefit of my experience for tender feet? I suffered fearfully, and was at great expense in getting all kinds of boots, still no relief. A friend advised me to try cachemire boots, and the effect was magical. This was five years ago, and ever since I have worn either boots or shoes and slippers of cachemire. Only in snowy or very wet weather do I venture on kid boots. The application of a mild solution of tincture of arnica (a teaspoonful of tincture to a full wineglass of water, gently dabbed on night and morning, allays any inflammation there may be. I have quite as much standing now as I ever had, and do not know what tender feet means.

### ROYAL BRITISH NURSES' ASSOCIATION.

Dr. Hugh Woods writes: In The Hospital of January 1st Dr. Bezly Thorne says that my account of the proceedings, which culminated in the granting of an *ex-officio* seat to the President of the Incorporated Medical Practitioners' Association on the Executive Committee of the Royal British Nurses' Association, is untrue. I presume he admits that an *ex-officio* seat was given in the bye-laws of the Nurses' Association to the President of the Incorporated Medical Practitioners' Association. Until Dr. Bezly Thorne gives a plausible explanation of the grounds for this step on the part of the Royal British Nurses' Association, I must ask the members of the Association to accept my deliberate statement that it was the result of negotiations between the Incorporated Medical Practitioners' Association and persons who were known to be representatives of the Nurses' Association, and who, as a matter of fact, got the above provision inserted in the bye-laws and approved by her Majesty's Privy Council. Whether or not these gentlemen acted "*ultra vires*" or "constituted themselves impostors" is for them to say.

### THE NURSING OF TYPHOID FEVER.

"H. B. H." writes: A short time ago a trained nurse of my acquaintance showed me an article on this subject which had appeared in The Hospital, and asked my opinion of the treatment there recommended by the writer, who is described as "A Trained Nurse." I mentioned several points on which I differed from the author of the article, and as the subject of typhoid is one which has occupied much of my time and attention, and which is always interesting to me, I should like much if you would allow me to state in your columns the points on which I think the advice given by the writer of the article in question is likely to mislead a nurse who follows it without using her own discretion. The essentials of foods suitable for typhoid cases are: (1) They must be nutritious, light, fluid; (2) non-gaseous, nor fermenting, nor likely to ferment in the stomach; (3) non-stimulating, except where the pulse flags and the strength gives way; (4) of a temperature between 60° and 80° Fahr., if possible. Now, ice-cream, which is strongly recommended in the article in The Hospital, must, to be suitable for a typhoid patient, be home-made, and consist only of cream, flour or arrowroot, water, and a trace of sugar. The idea of using it in typhoid is not at all new, but it has never become popular because it must not be swallowed cold. The necessary apparatus is not always at hand. "Heady" cases bolt it down at once. It is only desired during the feverish stage, and then it is that patients are "heady." If swallowed cold it is really dangerous, because the local anæmia produced in the stomach and bowel is followed by reactive hyperæmia, and so the danger of hæmorrhage from an ulcerated surface is increased. So, too, gaseous distention of the bowel must be avoided, and this necessitates aërated waters being allowed to stand till they are no longer aërated; it also bars koumiss (which continues to ferment in the stomach); and champagne should be given flat. Then the writer advises the uses of Valentine's meat juice with sherry, which would be almost certain to repeat and cause acid regurgitations, particularly with Marsala and sweet sherries. Valentine is all right by itself or in water. I prefer it as a stand-by in the late stages as a stimulant. The writer seems to give stimulants all through, which is bad practice. Spirits are to be preferred to wines in typhoid; in fact, I bar all wines except the best of dry champagne, and allow that only flat, and when spirits—(1) whisky, (2) brandy—are badly borne. Yolk of egg is wrong, as it is digested in the duodenum, which has to repair its ulcers, and so has quite enough to do. White of egg, however, is good, and is digested in the stomach. The "trained nurse" says nothing of the straining of soups, nor of the temperature at which food should be administered; and she omits all mention of the lighter forms of farinaceous diet in convalescence—barley water, rice water, home-made beef juice, and Carnrick's peptonoids. Nay, she does not seem to know that antiseptics are given to keep down fermentation and its resulting flatulent distention. The article throughout shows a lack of experience and an absence of that precision and detail which one expects in an honest treatment of a serious subject. I must apologise if my criticism is less kind than a lady writer might expect; but my desire to be courteous must give way to the necessity of speaking what I believe to be the truth on a subject of such importance.

### BED SORES.

Sister White writes: The best thing to prevent bed sores is to keep the bed-clothes quite smooth and dry. Wash the skin with soap and warm water night and morning, dry thoroughly, dust with zinc or boric powder, then rub with brandy or methylated spirits, and, if possible, put your patient on a water pillow or air cushion. If you cannot get this small pads with holes in them to relieve the joints of pressure are good. I have found zinc or boric ointments when the skin is looking red very valuable. I have never used alum water, nor have I had to powder my starch or charcoal, but I have now done some with a sheet of paper and a flat-iron. Put the charcoal or starch in a double piece of paper, then roll it with a wringer stick or crush it with an iron. I do not think you will find it much trouble, and I hope it will be satisfactory. I should not mind doing it myself.

"A Practical Nurse" writes: In answer to your correspondent "M." on the question of bed-sores, may I give a little of my practical experience which has been varied during my nursing career, consisting of ordinary cases, neglect, paralysis, old age, various fevers and cases which have been brought in from sea, lying disabled in their dirty wet clothes for five or six days with a crushed pelvis, compound fractures, &c., aggravated by the continual pitch and toss of a fishing smack or steam trawler in dirty weather. Now I cannot speak theoretically on this subject, but as a pro. I had to "do backs" twice a day, whether they wanted it or not. After the manner of your querist, we first had to wash them with warm soap and water, then we rubbed them with methylated spirit, and finally dusted them with zinc or starch powder. Our sisters used to tell us to tell us to rub them well, with a steady, gentle pressure and the palm of the hand, so as to assist the circulation of the blood, and twice a week we used zinc ointment to prevent the skin from cracking. As every one knows, a bed-sore is considered a reflection on a nurse, though at times they will come. When a back becomes inflamed I have found that to paint the parts with solution of silver, once or twice if necessary, a good remedy. One sister I worked with used stiptic collodion, the edge of which was apt, however, to curl, and often caught in the crease of a garment or on the edge of a vessel if a young or busy nurse was giving the necessary attention; and in old people especially it was liable to pull the loose skin with it. In hospitals or the houses of wealthy patients one can generally get an air or water cushion; when these are not obtainable the position of the patient should be constantly changed when practicable. If not, rings or pads should be made of old clean linen, wool, or even fine tow covered with linen or muslin, but on no account strapped on to the patient as I have often seen done. In wet cases the patients should be kept well greased in the groin and on the buttocks with zinc ointment to prevent in a great measure the action of the urine on the skin. Personally, as soon as the skin shows signs of cracking, I notify the fact to the doctor in charge, as it is a mistake to wait for it to break. A doctor generally knows a conscientious nurse when he has one to deal with, and he will think far more of her if she reports it than to find out after the back is broken; besides, he may probably know of a remedy to prevent it which even the oldest nurse may not have heard of. After the skin is broken the remedies are numerous—zinc lotion, boracic ointment, zinc ointment, and vinolia cream are the most common. Should it be a sloughing sore, poultices and fomentations are often used to clean them up.

### A DISTRICT NURSE'S PAY.

F. TAYLOR writes: Will you allow me to reply to the nurse's letter, in which she says she has £65 a year, but "can make no provision for the future," though she makes "both ends meet." My income is the same, and I save ten shillings a week out of it. In my last district it was one pound a week, and I saved five shillings a week. Does she mean to hold out her hand for charity in her old age, to eat its bitter bread? Let me assure her that though good days may come, as the skies may fall, bad days will come, as we are all mortal, and our lives must fail toward the end. I have lived for years in France, and the working people out of every franc earned put some sous aside to preserve their independence. They would consider it madness to make no provision for the rainy day. Nothing could be more suicidal than the improvidence and want of forethought of the English colony where I worked in comparison to the French natives. I, who through the foolish indulgence of parents had to learn the hard lesson of economy after I had grown up, beg E. G. to consider how many homes she must see in her daily work where nothing like £65 a year is earned. I have seen needy gentlemen out at elbows in garrets whose wants several hundreds a year would not have satisfied at one time, yes, and dying alone in the dreadful solitude of a cadging poverty. Let her say to herself, "As they are, so may I be, unless I take heed now that I can work." And how much more easily an old woman is provided for comfortably than a man? Excuse my taking the liberty of troubling you, but I feel deeply on the subject. People who have to work should learn to be their own best friend.

*** We should like to have headed Nurse Taylor's letter "Thrift," under which title we could endorse every word she has written. However, as she writes in reference to "A District Nurse's Pay," whilst agreeing with her as to the urgent need of all to be thrifty and to ensure their independence whatever sum they may earn, we nevertheless hold that it is the worker's bounden duty, and especially the woman-worker's duty, to fit herself thoroughly for whatever calling she may choose, and to demand fair pay. For a nurse to save is one thing, for the public to save at the nurse's expense is quite a diff rent thing.—ED. *T.H.*

### NURSING THE PLAGUE AT POONA.

THREE NURSES FROM THE GENERAL PLAGUE HOSPITAL, POONA, write: Having seen in the *Nursing Record* of November 20th a letter written by a lady in Poona, who has gathered a few erroneous ideas about our work here, we think it only fair to give our friends at home a short account of the true state of things here.

The hospital, or camp, is composed of 36 wards and about 40 tents for convalescent and observation cases. The wards are merely sheds with mud floors, whitewashed inside and out. Each is lined with rows of very roughly-made wooden cots, and has a small medicine table and a box for clothing and bedding. Each patient is allowed one sheet, one pillow, and three blankets. The wards look very bright in the daytime, with the red blankets against the white interior.

Each sister has charge of four or five wards, and under her she has ayahs and ward-boys. The work is very trying, the nursing naturally being done under great difficulties, not the least of them being the inability to understand the language. Natives are very dirty in their habits, and it is very difficult to make them use common sense or listen to reason.

The hours on day duty are eight, and on night eleven; the latter is most trying and ghastly work. Not only have we to contend with the horrors in the wards, but there is also the fear of snakes and jackals as we go about the camp quite alone with only a small hand lantern to light us. Luckily we only have to do it a week at a time.

The pay, while good, is by no means more than is required to meet our expenses, and we find it more difficult to save here than at home. Besides having to find food and servants, there are other expenses such as dress and carriage hire, for it is impossible to walk in the daytime, to say nothing of five rupees a month being taken for income-tax. We are lucky in being placed in such a nice station as Poona, where we have many advantages. Our quarters are very comfortable and people are very kind. We are most fortunate in having to work under such an admirable man as Surgeon-Captain Lloyd-Jones, who does everything he possibly can to further our comfort and happiness.

### Notes and Queries.

**The contents of the Editor's Letter-box have now reached such unwieldy proportions that it has become necessary to establish a hard and fast rule regarding Answers to Correspondents. In future, all questions requiring replies will continue to be answered in this column without any fee. If an answer is required by letter, a fee of half-a-crown must be enclosed with the note containing the enquiry. We are always pleased to help our numerous correspondents to the fullest extent, and we can trust them to sympathise in the overwhelming amount of writing which makes the new rules a necessity. Every communication must be accompanied by the writer's name and address, otherwise it will receive no attention.**

*An Asylum Publication.*

(112) Is there any weekly or monthly paper published in connection with asylum work?—*A Learner.*

The *Asylums News* is published at the County Asylum, Lancaster.

*A New Idea.*

(113) I have an idea for a new surgical appliance for taking off pressure, and therefore relieving pain. I have been a nurse some time, and have found it to be a great want. Could you tell me of anyone who would help me to bring it before the public, as I have no money, and find it would take £4 to register, and would it be safe to show till it has been?—*Auntie.*

We have been asked a similar question several times, and can only advise the inventor to try and get a respectable firm to take "the idea up." Before any money is spent upon it, we would suggest that the opinion of some hospital surgeon be asked in regard to its utility. The etiquette of the medical profession is entirely opposed to patents being taken out by medical men for medical appliances, and we think "Auntie" would be quite safe in showing her invention to any surgeon of good standing.

*Congenial Work for Women.*

(114) May I ask further particulars as to the prospect of work in this direction? What qualifications are necessary, and to whom should application be made?—*Nita.*

There are few particulars to be given just at present. One or two Boards of Guardians are making experiments in this manner. We think that a three years' nurse's certificate will probably be necessary as the appointments will be made by the Poor Law Authorities. Applications will have to be made to the Board of Guardians who have to make the appointment.

